# Antigen processing machinery deficits in head and neck cancers: mechanisms of immune evasion and strategies for therapeutic restoration

**DOI:** 10.3389/fonc.2026.1907861

**Published:** 2026-07-22

**Authors:** Poorvi Iyer, Brendan L. C. Kinney, Brianna Brammer, Maxwell Y. Lee, J. Walker Rosenthal, Nicole C. Schmitt

**Affiliations:** 1 Department of Biochemistry and Molecular Biology, University of Georgia, Athens, GA, United States; 2 Department of Otolaryngology – Head and Neck Surgery, Emory University, Atlanta, GA, United States; 3 Winship Cancer Institute, Emory University, Atlanta, GA, United States; 4 Emory University School of Medicine, Atlanta, GA, United States; 5 Department of Otolaryngology – Head and Neck Surgery, Stanford University, Palo Alto, CA, United States

**Keywords:** antigen processing machinery, head and neck cancer, HLA class I, immunotherapy resistance, oral cancer

## Abstract

The efficacy of T-cell based immunotherapies relies on the presentation of tumor-specific antigens via the major histocompatibility complex (MHC) class I pathway. In head and neck squamous cell carcinoma (HNSCC), most patients display primary or acquired resistance to these therapies, many failing to derive long term clinical benefits. While the presence of antigen processing machinery (APM) deficits is well documented, the relative contribution of transcriptional, epigenetic, and post translational mechanisms to APM regulation remains a growing area of investigation. This review provides a synthesis of the structure of the APM and the driving factors, ranging from genetic mutations to regulatory networks, that may contribute to its deficit in HNSCC. We explain how oncogenic signaling and environmental stressors may take part in the degradation and epigenetic silencing of the MHC-I pathway, limiting the predictive utility of biomarkers like PD-L1. By evaluating the impact of tumor heterogeneity and demographic disparities on APM integrity, we look at several therapeutic strategies to restore tumor visibility. Ultimately, these evasion strategies provide a framework for developing personalized, adaptive regimens to overcome immunotherapy resistance and improve clinical outcomes for HNSCC patients.

## Introduction

Head and neck squamous cell carcinoma (HNSCC) includes malignancies of the oral cavity, lip, pharynx, and larynx. This disease accounts for approximately 890, 000 new cases and 450, 000 deaths annually with incidence projected to rise by 30% by 2030 (1). The main risk factors for HNSCC are exposures to carcinogens, such as tobacco and alcohol, and increasingly, high-risk human papillomavirus (HPV), which is linked to approximately 70% of oropharyngeal cancers (2, 3).

Despite standard therapy, nearly half of HNSCC patients experience disease recurrence (4). Anti PD-1 immune checkpoint blockade (ICB) using monoclonal antibodies such as pembrolizumab or nivolumab is the current standard of care for recurrent/metastatic (R/M) HNSCCs that express programmed death-ligand 1 (PD-L1). However, around 80% of patients with R/M HNSCC fail to respond or develop resistance, hence the need for strategies to overcome immunotherapy resistance (5, 6).

The efficacy of immune checkpoint inhibitors is dependent on the presentation of antigens to immune effector cells so the adaptive immune can recognize and eliminate cancerous cells (7). This process is carried out by the antigen processing machinery (APM): a network of molecular components responsible for the generation, transport, and loading of antigenic peptides onto major histocompatibility complex (MHC) molecules (8). In the HNSCC tumor microenvironment, low levels of cytokines like interferon-*γ* (IFN-*γ*) lead to downregulation of these components. Because APM is a interferon-inducible system, this lack of induction prevents the presentation of tumor-specific neoantigens to cytotoxic CD8+ T-cells (9).

Around 70% to 80% of HNSCCs exhibit this impaired APM expression, characterized by the reduced presence of key components such as TAP1 and MHC class I (also known as human leukocyte antigen or HLA in humans) (10). This downregulation is associated with poor survival outcomes and represent a form of resistance that bypasses the reach of established biomarkers like PD-L1 (10–12). By exploring our current understanding of APM structure including the genetic, transcriptional, and post-translational mechanisms driving its deficit, this review establishes a framework for understanding immunotherapy resistance in HNSCC and offers potential strategies for restoring tumor visibility.

## The antigen processing machinery: discovery, structure, and function

### The discovery of the antigen processing machinery

Early understanding of MHC began in the early 20^th^ century, where the genetic basis for tissue compatibility was studied through murine tumor graft rejection studies (13). By the mid 1940s, George Snell utilized congenic mouse strains formalizing the *H-2* locus as the primary genetic driver for immune self-recognition (14).

In the early 70s, Zinkernagel and Doherty demonstrated what is now known as MHC restriction, proving that CD8+ T-cells require the dual recognition of a foreign peptide and a self-MHC molecule (15). This discovery prompted increased investigation into intracellular processing, leading to the 1980s understanding that endogenous proteins are proteolytically cleaved into peptides before surface presentation (16).

In the 1980s and the early 1990s the 20S/26S proteasome and the transporter associated with antigen processing (TAP1/TAP2), which mediates the ATP-dependent translocation of peptides across the endoplasmic reticulum (ER) membrane, were identified (17, 18). Concurrently, the discovery of the peptide loading complex (PLC), including specialized chaperones (calnexin, calreticulin, ERp57, and tapasin), elucidated the regulated component events required for MHC class I stabilization and peptide selection (19). For the purposes of this review, we will be exclusively discussing APM related to the MHC class I pathway. While MHC class II presentation is also very important to the immune landscape, it uses distinct pathways and machinery that are beyond the intended scope of this discussion.

### Breakdown of APM: MHC class-I antigen presentation pathway

#### Proteasome (LMP2/LMP7)

**Figure 1:**
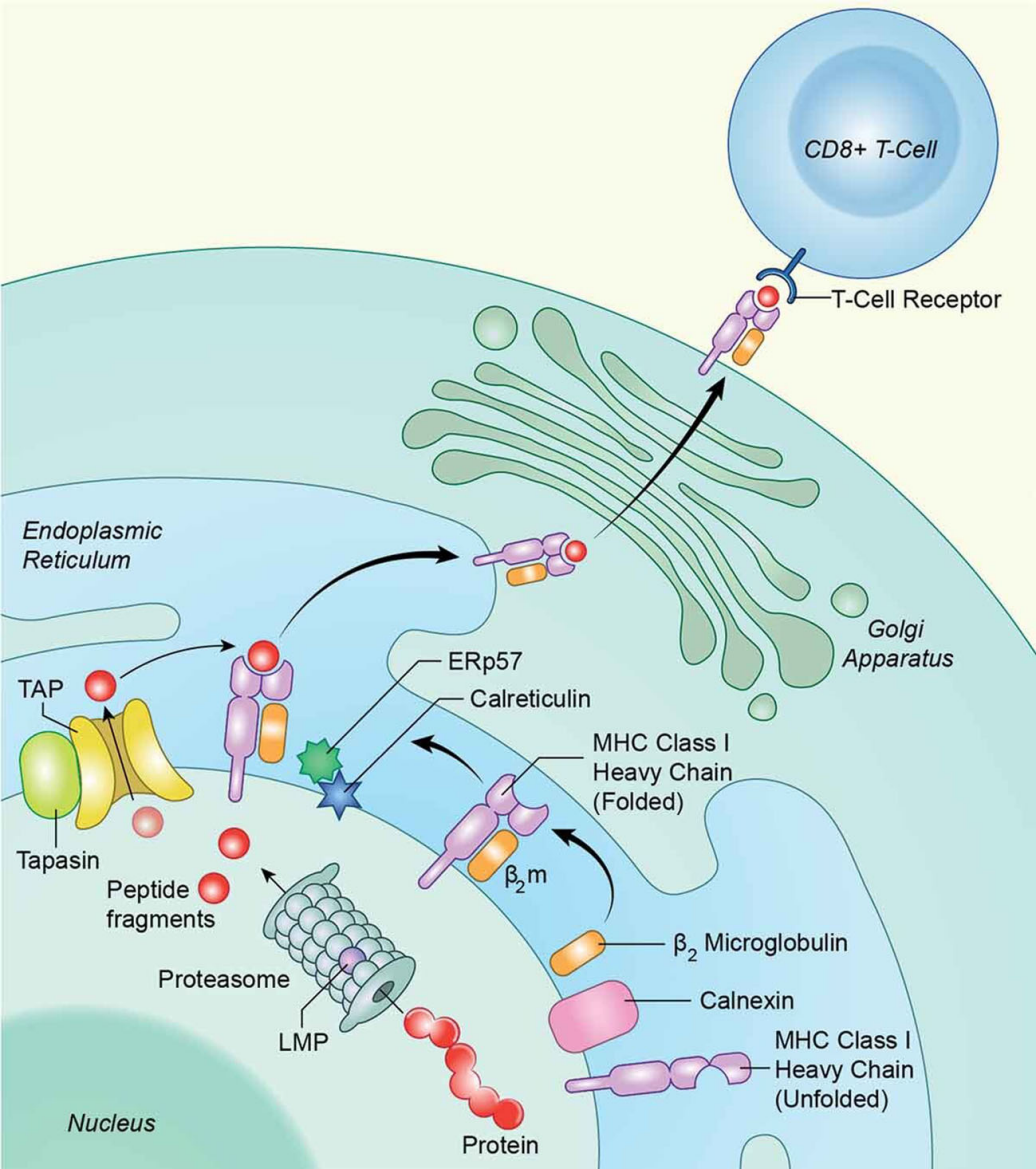
Illustration of the MHC class I antigen presentation pathway. Schematic diagram demonstrating the steps required for immunosurveillance, beginning with cytosolic proteasomal degradation, movement through the PLC within the ER lumen, and concluding with surface presentation to CD8+ T-cell. From Ye et al., 2020.

The generation of peptides to be presented on MHC class I begins in the cytosol with the degradation of endogenous proteins (**Figure 1**) a process mediated by an important network of molecular components (**Table 1**). Peptides presented include those involved in normal cellular proteins undergoing recycling, misfolded or damaged proteins, and aberrantly expressed tumor proteins.

**Table 1:** Functional roles of the MHC class I antigen processing machinery.

Component	Subcellular location	Functional role
LMP2 & LMP7	Cytosol	Catalytic β-subunits that form the immunoproteasome; optimize peptide cleavage for C-terminal residues that favor TAP affinity.
TAP1 & TAP2	ER Membrane	Heterodimeric (ABC) transporter that translocates cytosolic peptides into the ER lumen using ATP hydrolysis.
Tapasin	ER Lumen	A structure attached to the TAP complex that facilitates loading and sorting of peptides to MHC-I to prioritize high-affinity ligands.
HLA-A, -B, -C	ER → Surface	Polymorphic heavy chains whose α1/α2 domains form the peptide-binding groove.
β2-microglobulin	ER Lumen	Invariant light chain necessary for the structural stability and proper folding of the MHC heavy chain.
Calnexin	ER Membrane	Chaperone that stabilizes the newly synthesized, unfolded MHC I heavy chain. Responsible for initial folding and prevents aggregation before the chain association with β2M.
Calreticulin	ER Membrane	Chaperone that stabilizes the MHC-I heterodimer before peptide addition and facilitates glycan-dependent folding.
ERp57	ER Lumen	Thiol oxidoreductase that ensures correct disulfide bond formation; covalently linked to tapasin via a Cys95-Cys57 bond.

The 20S/26S proteasome is a large, barrel-shaped, catalytic protein complex that breaks down polyubiquitinated proteins into oligopeptide fragments (20). The core of the proteasome is a 20S, 700kDa cylindrical particle made of 28 subunits arranged into four heptameric rings. The outer rings, composed of seven *α*-subunits, have primarily structural functions, whereas the 7 *β-*subunits are the main contributors to catalytic activity. The 26S proteasome (1, 500 kD) contains the 20S components with an additional regulatory subunit (20, 21).

Under normal conditions, the proteasome is active. In immune cells or under inflammatory conditions, specifically in response to IFN-*γ* signaling, the proteasome undergoes modification to form the immunoproteasome. This transformation is distinguished by the replacement of catalytic *β*-subunits - *β*1 (*PSMB6*), *β*2 (*PSMB7*), and *β*5 (*PSMB5*) - with IFN-*γ* inducible subunits: *β*1i (*LMP2*), *β*2i (*MECL*-*1*), and *β*5i (*LMP7*) (22). The immunoproteasome often generates peptides with C terminal hydrophobic or basic residues, which are preferred anchor residues for MHC class I molecules (22). The proteasome produces peptides with a size distribution of 3–30 residues with an optimum of 6–11 residues (23, 24). This size corresponds in part to the size of antigenic peptides bound to MHC class I molecules (typically 8–10 amino acids), increasing likelihood of a specific T-cell response (25).

#### Transporter associated with antigen processing (TAP1/TAP2)

Following generation in the cytosol by the proteasome, the newly formed peptides must be transported into the lumen of the ER, where MHC class I molecules reside. This translocation step is carried out by the transporter associated with antigen processing (TAP) a member of the ATP-binding cassette (ABC) transporter subfamily B. TAP functions as a heterodimer composed of two distinct subunits, TAP1 and TAP2, which together form a translocation pore across the endoplasmic reticulum (ER) membrane (26). Each subunit has a transmembrane domain comprising multiple membrane spanning segments and a cytosolic nucleotide binding domain (NBD), which contains the conserved Walker A and B motifs (27).

Peptide binding to the cytosolic face of the transporter triggers a conformational change that couples ATP hydrolysis to the translocation of the substrate into the ER lumen. This process shows the most effective translocation for peptides 8–12 amino acids in length and possessing hydrophobic or basic C-terminal residues (17, 28).

The TAP1 NBD likely initiates the transport cycle, with ATP binding at this subunit and inducing subsequent ATP binding and hydrolysis at the TAP2 NBD (29). This sequential hydrolysis is a main determinant of immune surveillance, making it a frequent target for both viral evasion and oncogenic transformation. Downregulation or mutational loss of *TAP* genes is a well-documented mechanism of tumor escape, especially in metastatic breast and lung carcinomas where mutations between the C-loop and the Walker B motif can inhibit transport function (30). Reduced TAP expression results in a large deficit of surface presented MHC class I peptide complexes.

#### Peptide loading: calreticulin, calnexin, tapasin, and ERp57

The functional MHC class I molecule is a heterodimer composed of a polymorphic heavy chain (encoded by the *HLA -A*, *-B*, *-C* loci) and an invariant *β*2-microglobulin (*β*2M) light chain which is encoded on chromosome 15. The heavy chain features three extracellular domains *α*1, *α*2, and *α*3), with the *α*1 and *α*2 domains forming the specialized peptide-binding groove (31, 32). Association with *β*2M is non-covalent but is necessary for the structural stability of the heavy chain and its subsequent ability to bind antigenic peptides within the ER.

The assembly of the MHC class I molecule occurs in distinct stages within the ER. First, newly synthesized unfolded MHC class I heavy chains are stabilized by calnexin, a membrane-bound chaperone that ensures the chain folds correctly before it can dimerize with *β*2M. Once this initial heterodimer is formed, calnexin is released and replaced by calreticulin, a soluble homolog that facilitates further folding by specifically binding to monoglycosylated N-linked glycans on the nascent heavy chain (33, 34). ERp57, a thiol oxidoreductase, works alongside calreticulin and supports the correct formation and stabilization of disulfide bridges within the MHC class I heavy chain (35). At this stage, the molecule joins the peptide loading complex (alternatively known as the macromolecular loading complex), where tapasin acts as a bridging molecule that connects the TAP transporter to the MHC class I molecule. While the TAP complex pumps peptide fragments from the cytosol into the ER lumen, tapasin facilitates the loading and sorting of these peptides, replacing low-affinity ligands with high-affinity ones (36). Notably, some structural models demonstrate that tapasin and ERp57 are covalently linked by a disulfide bond between the Cys95 and Cys57 residues of tapasin and ERp57, respectively, forming a stable heterodimer (37).

#### Surface expression of MHC class I complex

A stable, high affinity MHC class I peptide heterotrimer is necessary for subsequent peptide mediated detachment of the complex from the loading machinery. Once released, these fully assembled and loaded complexes are sequestered into ER budding vesicles for export through the Golgi apparatus and the trans Golgi network to the cell surface through the secretory pathway (38). The display of these peptide MHC class I complexes on the plasma membrane of nucleated cells allows for their recognition by CD8+ cytotoxic T lymphocytes (CTLs). By identifying and binding to cells presenting foreign or aberrant peptides, CTLs initiate a potent adaptive immune response, leading to the elimination of the compromised cell through induced apoptosis or lysis (9). The polymorphism of the human HLA system across the HLA-A, -B, and -C loci, determines the repertoire of antigenic peptides an individual can present (39). Despite structural homology of these HLA molecules, their specific binding groove sequences are associated with different peptide affinities and expression levels, with HLA-A and HLA-B typically being the main targets for T-cell recognition (40, 41). This diversity is important for the development of cell-based immunotherapies; for example, many TCR-engineered T-cell constructs, including those targeting the HPV-16 E7 oncoprotein, are restricted to the most common alleles such as HLA-A*02:01 to maximize population reach (42).

## APM deficits in HNSCC

### APM in HNSCC

Defects in the antigen-processing machinery (APM) are a hallmark of immune evasion in head and neck squamous cell carcinoma (HNSCC). A 2005 study conducted by Meissner et al. demonstrated that treatment naïve tumors frequently do not express detectable APM, occurring in up to 70% of HNSCC cases. Specifically, their immunohistochemical analysis revealed significant deficiencies in key subunits: LMP2 and LMP7 were downregulated in 72% and 44% of lesions, respectively, while the peptide transporters TAP1 and TAP2 showed defects in over half of the samples studied. Additionally, it was identified that chaperones such as calnexin and ERp57 were downregulated in approximately 25% of laryngeal lesions, further compromising the assembly of the HLA class I complex (10, 11). This impairment in antigen presentation correlates with reduced infiltration of CD8+ T-cells within the tumor microenvironment and the creation of a “cold” tumor phenotype characterized by immune evasion (43, 44).

### Genetic and epigenetic alterations

**Figure 2:**
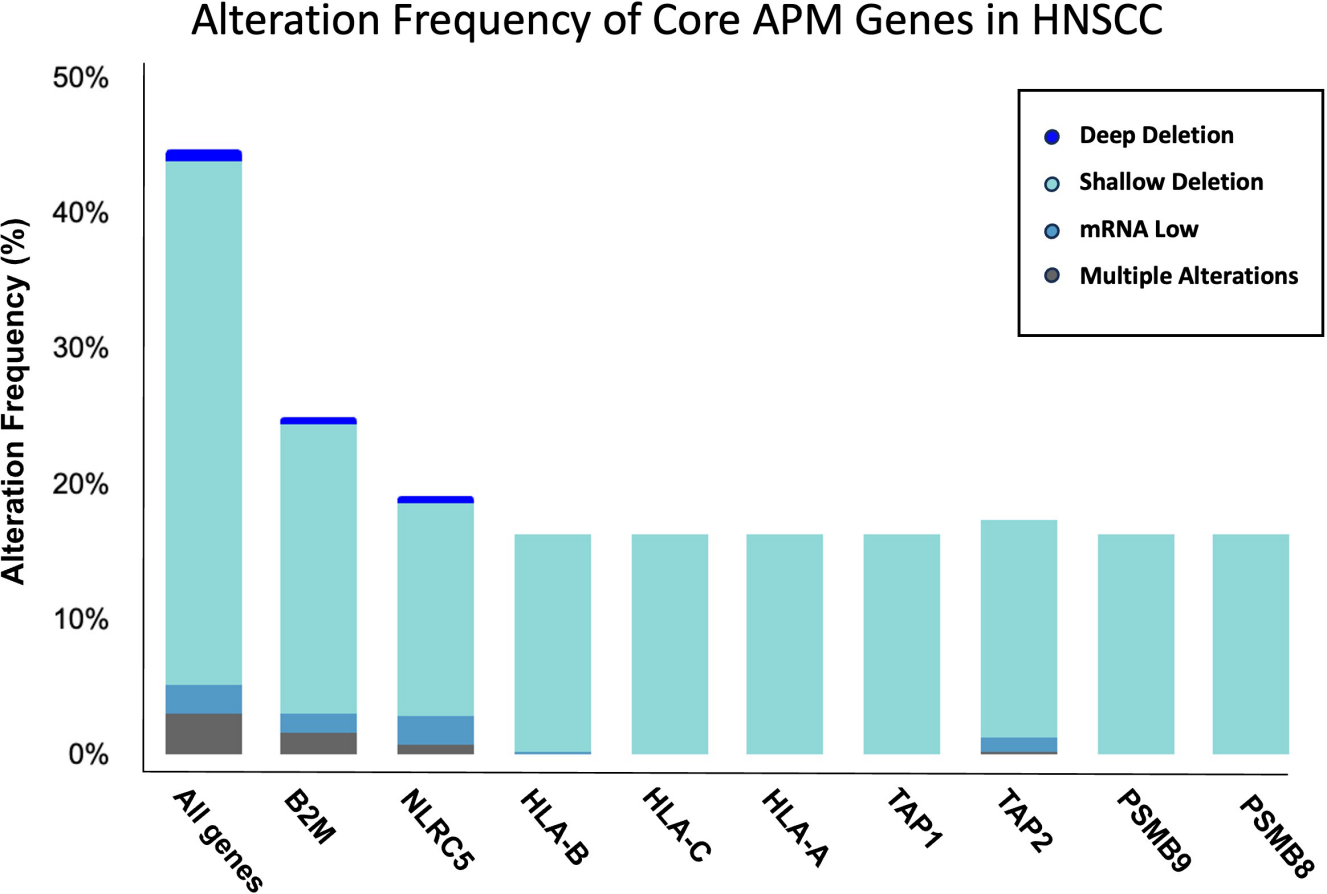
Alteration frequencies of core APM genes in HNSCC. Analysis of the TCGA Pan Cancer Atlas cohort (n = 488 complete samples) analyzed using whole-exome (mutations/CNA/structural variants) and RNA-seq (transcriptomics) data identifies a ~45% pathway deficit. Genomic loss via GISTIC 2.0, including deep deletions (dark blue) and shallow deletions (light cyan), represents a primary mechanism of evasion. Transcriptomic downregulation (light blue) was defined by a Z-score threshold < -1.5 (RNA-seq) relative to diploid samples. Dark grey denotes multiple co-occurring alterations. Analyzed genes include *β2M, NLRC5, HLA-A/-B/-C, TAP1/2, and PSMB8/9*. Data sourced from cBioPortal.org.

The disruption of the APM in HNSCC arises from a combination of genetic lesions and epigenetic modifications. Some deficiencies result from somatic mutations or loss of heterozygosity in critical genes such as *TAP1, TAP2, β2M* and the *HLA class I* heavy chains. However, Meissner et al. demonstrated that APM abnormalities in HNSCC are mainly attributable to transcriptional downregulation rather than structural gene abnormalities, as several defects could be restored through IFN-*γ* treatment (10). Analysis of the HNSCC Pan-Cancer The Cancer Genome Atlas (TCGA) cohort (n=488) demonstrates that 45% of patients harbor significant genomic alterations or mRNA downregulation in core APM components (**Figure 2**). The *NLRC5* promoter is frequently hypermethylated in various cancers, and this methylation level is negatively correlated with the expression of MHC class I genes and patient survival (45, 46). Furthermore, Zhou et al. (2020) utilized TCGA data from 522 HNSCC tumors to demonstrate that the enhancer of zeste homolog 2 (EZH2) methyltransferase triggers H3K27me3-mediated silencing on the promoters of *HLA -A*, *-B*, *-C, β2M*, and *TAP1* (47).

### Transcriptional control

#### IFN-γ and STAT1

The expression of APM components requires strict transcriptional control, mainly through the IFN-*γ*/JAK/STAT signaling. Activation of STAT1 is very important for this process. Upon phosphorylation at the tyrosine residue (pSTAT1 Tyr701), STAT1 dimerizes and translocates to the nucleus, where it binds specifically to gamma-activated sequences (GAS) within the promoters of APM component genes and activates transcription of *HLA* class I molecules, *TAP1*, and immunoproteasome subunits. However, a feature of HNSCC lesions is a low STAT1/pSTAT1 ratio. Leibowitz et al. established that this deficiency of activated STAT1 directly mediates TAP1-dependent escape from recognition by tumor antigen specific CTLs, contributing to a “cold” tumor phenotype in HNSCC patients (48, 49). HNSCC tumors lacking functional STAT1 demonstrate impaired antigen presentation, poor recognition, and CTL escape, suggesting this node as a viable target for APM rescue (50). Furthermore, a recent study noted that aberrant phosphoinositide 3-kinase (PI3K) signaling directly interferes with IFN-*γ*-pSTAT1 axis and, in turn, inhibits MHC-I (51).

It is important to note that type-I interferon (IFN-I) plays a role in MHC class-I regulation. HPV E5 protein may inhibit the expression of the IFN-I pathway by binding to stimulator of interferon genes (STING), thus restricting the immunoproteasome and in turn, the MHC-I repertoire in HNSCC (52). Saturated fatty acids have interestingly shown to also be a potential STING-IFN-I inhibitor (53).

#### SHP2

**Figure 3:**
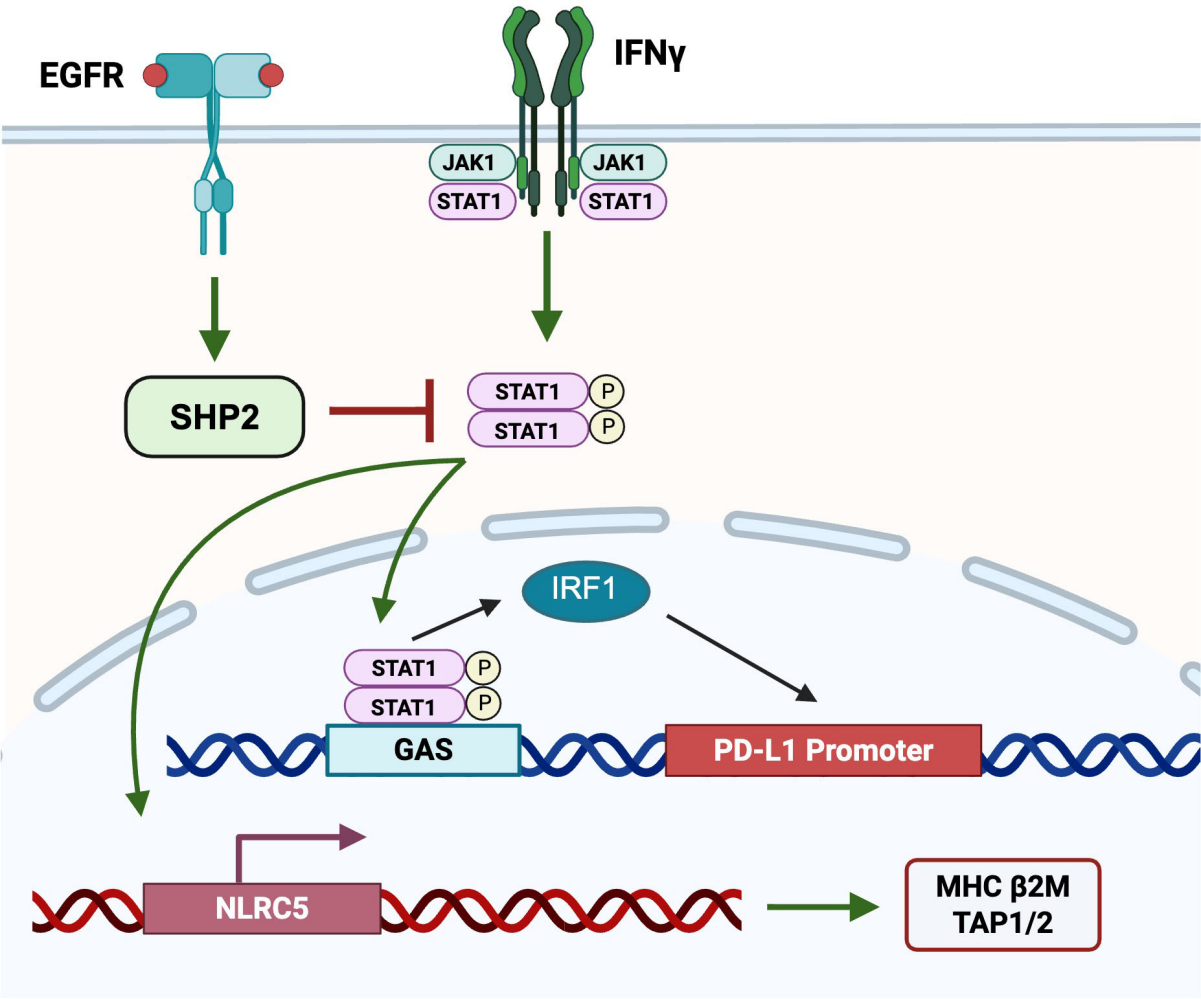
Schematic of convergent signaling pathways regulating the MHC class I antigen presentation pathway. IFN- γ signaling induces NLRC5 and PD-L1 via pSTAT1, whereas EGFR/SHP2 signaling inhibits this axis to suppress tumor visibility and drive immune evasion in HNSCC. Created with biorender.com.

The Src homology 2 domain-containing phosphatase 2 (SHP2, encoded by *PTPN11*) serves as a negative regulator of the pSTAT1-mediated antigen presentation pathway in HNSCC. The characteristic overexpression and constitutive activation of the epidermal growth factor receptor (EGFR) in HNSCC specimens facilitate the recruitment of SHP2 to phosphotyrosine docking sites on the receptor. Upon activation downstream of EGFR, SHP2 catalyzes the dephosphorylation of pSTAT1 at both the Tyr (701) and Ser (727) residues. This SHP2 mediated dephosphorylation effectively terminates STAT1 transcriptional activity, inhibiting expression of downstream APM components (54). The interaction between the proinflammatory IFN-*γ* axis and the inhibitory EGFR/SHP2 pathway is illustrated in **Figure 3**.

#### NLRC5

NLRC5 (nod-like receptor family caspase recruiting domain-containing 5), also known as MHC class I transactivator (CITA), functions as a transcriptional activator specifically for MHC class I genes and APM components (**Figure 3**). NLRC5 does not bind DNA directly but instead interacts with MHC class I promoters to form a transcriptional enhanceosome (46, 55, 56). This interaction drives the expression of *HLA -A, -B, -C*, and *β2M*. Downregulation of NLRC5 activity leads to APM deficits. Notably, analysis of the HNSCC TCGA cohort demonstrates that *NLRC5* is dysregulated in approximately 20% of cases, predominantly through shallow deletions and reduced mRNA expression (**Figure 2**). These *NLRC5* transcript levels show a strong positive correlation (r_s_≈0.68, n=522) with HLA expression in HNSCC (46).

#### p53

Wild type p53 communicates with the adaptive immune system by directly upregulating proteins involved in the MHC class I antigen presentation pathway. Specifically, p53 binds to p53-responsive elements in the promoters of the *TAP1* and endoplasmic reticulum aminopeptidase 1 (*ERAP1*) genes to induce their transcription. ERAP1 acts as a molecular editor to trim N-terminal extensions of peptides to the optimal length of 8 to 11 amino acids for MHC binding (57, 58). *TP53* mutations, which occur in over 80% of HPV-negative HNSCC, result in decreased ERAP1 and TAP1 levels, leading to significantly lower surface MHC class I expression (57, 59). The dual loss of functional p53 and STAT1 signaling results in a “hard” APM deficiency where tumor cells are unable to ramp up antigen presentation even when exposed to immune stimuli (50).

### Post-translational mechanisms

Beyond transcriptional control, the APM deficit in HNSCC can also stem from post-translational mechanisms that impact the stability, proper folding, or accelerated degradation of APM components or MHC class I molecules themselves.

Malignant cells often reprogram cellular protein sorting to reduce surface MHC class I expression and antigen visibility. In HPV-positive HNSCC, the oncoprotein E6 induces the expression of the membrane-associated RING-CH-type finger 8 (*MARCHF8*) E3 ubiquitin ligase. While MARCHF8 is known to ubiquitinate MHC class I heavy chains for degradation, it also plays an important role in stabilizing the E7 oncoprotein. Specifically, MARCHF8 binds to and ubiquitinates CUL1 and UBE2L3 (components of the SCF ubiquitin ligase complex), targeting them for proteasomal degradation. By depleting these factors, MARCHF8 prevents the ubiquitination and subsequent degradation of E7, maintaining the high levels of E7 required for tumor maintenance (60).

Further studies explore increased expression of the long noncoding RNA *HIF1A-AS2* due to intratumoral hypoxia. Research indicates that HIF1*α* directly binds the regulatory region of *HIF1A-AS2* to upregulate its transcription. HIF1A-AS2 acts as a scaffold to facilitate the interaction between MHC-I and the autophagy cargo receptor NBR1. This complex recruits MHC class I molecules to autophagic vesicles for degradation (61).

### Genomic alterations and clinical significance

The integrity of the APM is a primary clinical determinant of whether a tumor maintains immunological “visibility” to CTLs. Data from clinical trials at the National Cancer Institute indicate that non-responders to TCR-engineered T-cell therapy targeting the HPV-16 E7 oncoprotein typically exhibit tumors with somatic loss or mutation of *TAP1/2, HLA-A, or β2M* (42). This suggests that while regulatory repression may dominate the pre-treatment tumor landscape as described by Meissner et al. (2005), genomic elimination of APM components is a viable escape mechanism under the selective pressure of adoptive cellular therapies. Clinically, the implications are severe; APM defects, particularly the loss of LMP7 are significantly associated with disease recurrence within a two-year post-treatment window and poor overall survival (p<0.05) in HNSCC patients (10). Furthermore, somatic HLA loss of heterozygosity (LOH) occurs in 53% of HNSCC cases, acting as a major driver of resistance to natural immunosurveillance (62).

### Common APM component deficit in other cancers

APM deficits are not unique to HNSCC but instead represent a common mechanism of immune evasion across a broad range of human malignancies. Downregulation of MHC-I has been reported in 40% to 90% of all human tumors (63). Importantly, many of these APM component losses are reversible through epigenetic or signaling modulation, providing a therapeutic opportunity for broader APM-restoring strategies.

## Impact of immunotherapy on APM and therapeutic strategies to restore function

### Impact of immunotherapy on APM

Modern immune checkpoint blockade (ICB) therapies, specifically those targeting the PD-1/PD-L1 axis, rely on a positive feedback loop to sustain antigen visibility. In a responding tumor, the successful reactivation of tumor infiltrating lymphocytes (TIL), specifically CD8+ cytotoxic T-cells, leads to a localized burst of IFN-*γ* (64, 65). This cytokine binds to the IFN-*γ* receptor 1/2 complex, activating the JAK/STAT signaling axis to drive the transcription of APM components, in turn presenting more antigens and further stimulating T-cell recognition and killing (66, 67). Importantly, this same IFN-γ/JAK/STAT signaling also drives the expression of PD-L1 itself (**Figure 3**) (68).

The failure of this loop characterizes a large portion of HNSCC patients who do not respond to ICB. In many “cold” tumors, the APM is essentially defective, stopping the initial T-cell recognition event and subsequent IFN-*γ* release. Evidence suggests that for tumors that cannot increase antigen presentation even when exposed to exogenous IFN stimulation, ICB alone is insufficient (12). Strategies aimed at restoring APM function, either before or concurrently with ICB, may convert the tumor into a state where effector cells will have a viable molecular target (10, 50, 69).

### The limitations of PD-L1 combined positive score

The programmed death-ligand 1 (PD-L1) combined positive score (CPS) is currently the most utilized clinical biomarker for selecting HNSCC patients for pembrolizumab, established by Burtness et al. in the landmark KEYNOTE-048 trial. CPS is calculated as the ratio of PD-L1-positive cells (tumor cells, macrophages, and lymphocytes) to the total number of viable tumor cells x 100 (4). While patients with a CPS ≥1 show improved survival outcomes using ICB compared to chemotherapy alone, the predictive response value of this score is limited (70).

Some investigators posit that this may be because PD-L1 is an indirect marker of strong upstream IFN-*γ* signaling rather than a direct measure of antigen presentation capacity (50) (**Figure 3**). PD-L1 can be upregulated independently of APM in some contexts, for example through STAT3 activation by lactate or specific oncogenic pathways (71). Furthermore, if there are downstream defects in the APM, the tumor may express PD-L1 while having low functional visibility to the T-cells (42).

The lack of the CPS biomarker to capture approximately 60% of resistant cases has driven interest in the STAT1/NLRC5/MHC signaling node as a more accurate biomarker of resistance. In clinical trials of neoadjuvant therapy, pre-treatment tumor cell HLA class I expression has been shown to be a more accurate predictor of pathologic response than traditional membrane staining of PD-L1. Spatial analysis of these tumors shows that T-cells predominantly colocalize with HLA class I-positive regions; therefore, areas of HLA loss act as blind spots and may allow the tumor to evade detection despite high T-cell infiltration (12).

Wang et al. addressed this limitation by developing the multi-omic Tumor Immunogenicity Score (TIGS) framework, which couples log-transformed mutational burden with a normalized 18-gene transcriptomic APM expression panel tracking *TAP1/2, β2M*, and HLA class I, among others. Use of TIGS evaluates both tumor antigenicity and antigen presentation efficiency, and has shown to outperform standalone TMB or PD-L1 tracking in predicting clinical response across high-signal cohorts like HNSCC (72).

However, we acknowledge that APM deficits explain only a subset of limitations regarding the use of PD-L1 expression as a biomarker. Because tissue-based PD-L1 immunohistochemistry does not reflect the circulating components of the immune landscape, recent studies explore liquid biopsies that track functional PD-1-binding soluble PD-L1 (bsPD-L1) alongside circulating matrix metalloproteinase. These enzymes enzymatically cleave membrane-bound PD-L1 and alter extracellular matrix integrity; in turn, this dual-marker approach, though currently characterized primarily in non-small cell lung and gastric malignancies, provides an interesting liquid biomarker profile that may more accurately predict ICB efficacy and tumor recurrence than staining in HNSCC (73, 74).

### Conventional modalities: chemotherapy, radiation, and targeted therapies

Historically, some standard-of-care treatments have been observed to partially rescue APM function. Mechanistically, this suppression of the immune response in HNSCC can be reversible; we previously demonstrated that cisplatin chemotherapy could enhance STAT1 activation by increasing phosphorylation at the serine residue (p-Ser727), thereby potentially restoring APM expression and re-sensitizing HNSCC cells to T-cell-mediated killing (75). Radiation therapy acts as a catalytic immunomodulator by inducing immunogenic cell death, which promotes the infiltration of CD8+ T-cells and a localized burst of IFN- γ, further upregulating MHC class I and other APM components to restore tumor visibility (76). EGFR is overexpressed in nearly all HNSCC cases and serves as a negative regulator of the APM via the recruitment of SHP2; thus, EGFR blockade with cetuximab (an anti-EGFR antibody) can trigger STAT1 reactivation and HLA upregulation (54). Zhang et al. (2022) discovered that the addition of cetuximab to PD-1 inhibitors significantly improved objective response rates specifically in patients with HPV-negative HNSCC (46% vs 15% for monotherapy, p < 0.001), but provided no benefit in HPV-positive disease. This may be in part because HPV-negative tumors tend to have more severe baseline APM deficits driven by oncogenic EGFR signaling, which can be specifically rescued by targeted blockade to unblock the STAT1/HLA pathway (69).

However, use of these conventional treatment modalities for APM restoration are accompanied by high toxicity, and there is potential for these treatments to inadvertently damage the very immune effector cells needed for tumor clearance (77). This has necessitated the exploration of more targeted, less toxic molecular restoration strategies.

### Targeted molecular restoration of APM

Clinical experiences with high dose interferon, notably within the Sunbelt Melanoma Trial, established that while systemic IFN signaling can improve relapse-free survival in melanoma, its therapeutic utility remains constrained by high systemic toxicity and a failure to extend overall survival (78). These findings show a need for targeted methods that bypass IFN dependencies to restore localized immune recognition and MHC-I mediated surveillance.

**Figure 4:**
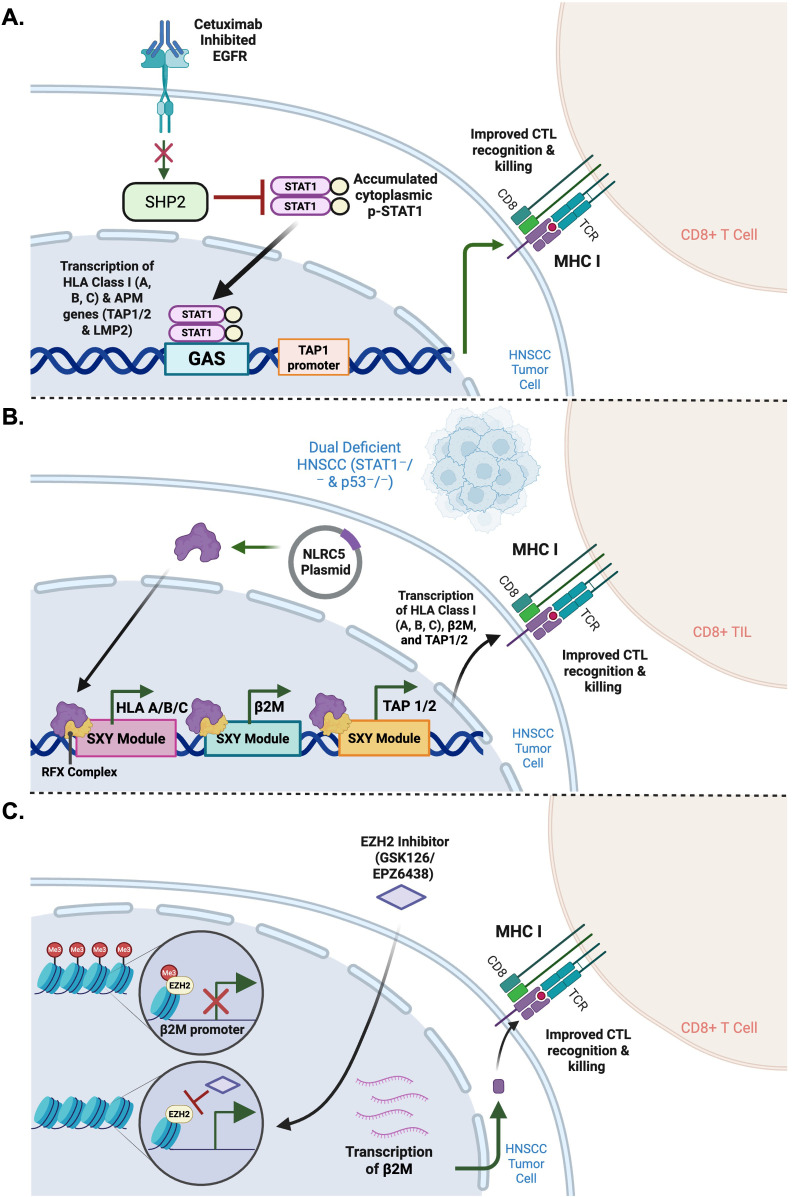
Mechanistic strategies for restoring MHC class I antigen presentation in HNSCC. **(A)** Restoration of the SHP2/STAT1 Axis. Monoclonal antibody blockade of EGFR (Cetuximab) inhibits SHP2 phosphatase activity, allowing for p-STAT1 nuclear translocation and APM gene transcription (Adapted from Srivastava et al., 2015). **(B)** Plasmid transfection of *NLRC5* bypasses signaling deficits to initiate *HLA-A, -B, -C, β2M*, and *TAP1/2* transcription. (Adapted from Kinney et al., 2024). **(C)** Epigenetic Reversal of *β2M* Silencing. Inhibition of EZH2 (GSK126/EPZ6438) reduces H3K27me3 marks, restoring *β2M* promoter accessibility/expression (Adapted from Zhou et al., 2020). Created with biorender.com.

Modern strategies now target these specific evasion points, such as through SHP2 inhibition. Preclinical evidence suggests that the inhibition or depletion of SHP2 specifically in HNSCC cells increases nuclear pSTAT1 accumulation and restores the expression of HLA class I molecules, thereby increasing the secretion of chemokines (**Figure 4A**) (67). A recent study demonstrated that PI3K inhibition enhances IFN-*γ* mediated induction of MHC class I and II by increasing STAT1 levels and genomic accessibility (51). In cases where upstream nodes like JAK or STAT1 are irreversibly lost, a more direct strategy involves restoring the downstream transactivator, NLRC5. Kalbasi et al. (2020) demonstrated that MHC class I presentation could be induced in JAK1-deficient melanoma cells via plasmid transfection of NLRC5, leaving them sensitive to adoptive cell therapy; a strategy we later validated in HNSCC (50, 79). Because NLRC5 operates downstream of STAT1, its restoration represents a viable strategy for bypassing upstream signaling blocks, such as those caused by SHP2-mediated dephosphorylation in HNSCC (**Figure 4B**) (50).

Beyond traditional signaling pathways, several novel therapeutics are emerging to bypass traditional immune dependencies. For instance, although primarily characterized in melanoma models, the intratumoral delivery of BO-112 (a nanoplexed double stranded RNA) mimic activates sensors like TLR3 and PKR to induce MHC-I expression via the NF*-*κB pathway (79). Like EGFR inhibition, small molecule inhibitor of apoptosis protein (IAP) antagonists, such as ASTX660 (tolinapant), may offer a safer, targeted alternative to the systemic toxicity of standard cisplatin-based therapies. Preclinical models show that while cisplatin can enhance STAT1 activation by increasing phosphorylation at the serine residues, its potential to also damage the very immune effector cells needed for tumor clearance limit its utility. ASTX660 sensitizes tumor cells to TNF-α, induces immunogenic cell death (ICD), and upregulates surface HLA-A, -B, -C expression (80). Unfortunately, IAP antagonists paired with chemoradiotherapy have also recently been shown to enhance growth of distant metastatic disease, limiting their potential for use in HNSCC (81).

Alternatively, the immunoproteasome represents another target for therapeutic upregulation, with recent data in laryngeal and hypopharyngeal malignancies demonstrating that the catalytic subunit β5i (LMP7) can be induced by ionizing radiation. This radiation mediated rescue relies on upstream non-receptor tyrosine kinase signaling to alter pre-mRNA processing in favor of the functional E2 splicing isoform, reversing presentation deficits (82).

Epigenetic strategies can reverse immune evasion by targeting specific histone modifiers; for example, LSD1 inhibition increases *H3K4me2* levels to upregulate MHC-I and stimulate CXCL9-mediated T-cell recruitment (83). Similarly, EZH2 inhibition via GSK126 or EPZ6438 reactivates the APM by reversing *H3K27me3*-mediated silencing of the *β2M* promoter, enhancing antitumor immunity and overcoming anti-PD-1 resistance (**Figure 4C**) (47, 84). Further, targeted suppression of *HIF1A-AS2* and inhibition of the autophagic pathway can successfully rescue MHC-I surface expression (61).

Finally, natural killer (NK) cell-based therapies are an interesting strategy for treating HNSCC tumors with major APM deficits, as they function independently of typical MHC-restricted presentation (85). This potential is shown by PD-L1 CAR-engineered NK cells, which eliminate HNSCC T-cell escape variants that upregulate PD-L1 following exposure to T-cell-derived IFN- *γ* (86). Also, the finding that IL-15 drive circulating NK cells toward an aggressive ieILC1-like phenotype provides another strategy for bypassing the antigen presentation failures that often leave normal T-cells ineffective (86, 87).

## Challenges/future directions

HNSCC is characterized by significant inter- and intratumoral heterogeneity, which poses a major challenge to the uniform application and efficacy of APM restoration strategies (88). Consequently, this spatial heterogeneity implies that a single therapeutic approach targeting the APM may not be effective across the entire tumor or in all patients (12).

The dynamic nature of APM expression and the TME also means that resistance mechanisms are not static and can evolve under therapeutic pressure. This necessitates dynamic, adaptive therapeutic approaches rather than a universal strategy (89). Addressing this complexity may require advanced profiling techniques like spatial transcriptomics and multiplex imaging to capture the intratumor spatial heterogeneity of MHC-I and APM expression. Utilizing these approaches is important for precise biomarker interpretation, ensuring that localized hot phenotypes are not obscured within an overall cold tumor categorization derived from bulk expression signatures, thereby providing a more ideal method for patient stratification (90).

### Optimizing delivery

For APM therapies involving gene delivery or specific molecular modulators, efficient and tumor-specific delivery methods are important. Developing delivery platforms that can effectively penetrate the complex and sometimes dense HNSCC tumor microenvironment to reach a sufficient number of cancer cells, while also minimizing toxicity, remains a significant challenge; however, a few mechanisms have demonstrated promise.

As previously discussed, to bypass IFN signaling defects, Kalbasi et al. (2020) utilized lentiviral delivery of the full-length *NLRC5* transactivator. This genetic approach successfully restored the complete MHC-I enhanceosome and critical components sensitizing JAK1 deficient tumors to adoptive cell therapy. Recent protein engineering, validated in melanoma and lymphoma models, have addressed the potential translational challenges posed by the large size of wild-type NLRC5 (approx. 205 kDa). Researchers have developed a more compact and efficient fusion protein, NLRC5-CIITA fusion protein (NLRC5-SA) roughly half the size of the 205kDa construct, which outperforms the wild-type version in preclinical studies, restoring MHC-I surface expression and promoting antitumor immunity (91). In regard to the IFN-I axis, a recent study demonstrates the use of manganese-coordinated nanoparticles to drive STING and IFN-I signaling, utilizing a novel metalloimmunotherapy mechanism to enhance anti-tumor immunity (92).

### Better understanding patient populations

A major obstacle to the uniform implementation of APM restoration is the significant molecular and demographic diversity within HNSCC cohorts, with different populations exhibiting different molecular drivers of immune evasion. Demographic factors also influence APM integrity; Black patients often present with lower intratumoral infiltration of effector immune cells (including CD8+ and γ𝛿 T-cells) and shorter survival compared to White patients (93).

Further, studies demonstrate that female HNSCC patients exhibit heightened T-cell activation and increased production of pro-inflammatory cytokines like IFN-*γ* and TNF-*α.* High-resolution transcriptomic profiling also confirms that female immune subpopulations feature basal upregulation of interferon-stimulated genes and an enrichment for core antigen presentation pathways (94). Mechanistically, because the APM is an interferon-inducible system, this combined priming and elevated local cytokine profile sustain the upstream JAK/STAT1 signaling axis in females, promoting the continuous transcription of core APM components. In contrast, male patients demonstrate increased T-cell exhaustion markers such as PD-1 and TIGIT, and a higher frequency of immunosuppressive myeloid derived suppressor cells (MDSCs) necessitating sex-informed restoration strategies (95).

Although novel non-invasive tools such as exosomal RNA profiling are being developed, further research is required to improve monitoring and characterization of APM states (96). Integrating these demographic and molecular variables will allow for more personalized regimens that maximize efficacy.

## Conclusion

APM deficits are a common mechanism of immune evasion in head and neck cancer. This escape mechanism contributes to the observed resistance against current immunotherapies, including ICB, despite the progress made in HNSCC treatment. PD-L1 primarily reflects the presence of an immune-inflamed microenvironment and intact upstream IFN-γ signaling, but it does not directly assess the critical functionality of the APM. APM status offers an important layer of information for patient stratification, explaining the resistance seen in several PD-L1 positive tumors.

APM deficits in HNSCC arise from genetic, epigenetic, transcriptional, and post-translational mechanisms. The recognition that many of the deficits in HNSCC are reversible, particularly those driven by epigenetic alterations or dysregulated oncogenic signaling, opens several therapeutic avenues. Strategies aimed at restoring APM function, such as signaling pathway inhibitors and other approaches to enhance IFN-γ signaling, hold potential.

Many groups are exploring combination strategies that integrate APM-restoring agents with existing immunotherapies. By simultaneously enhancing the tumor’s visibility to the immune system and enhancing T-cell activity, a more robust and sustained anti-tumor immune response may be achieved, potentially overcoming resistance and improving clinical outcomes for HNSCC patients. However, translating these promising approaches into clinical practice necessitates addressing challenges such as tumor heterogeneity through personalized, targeted delivery. Ultimately, understanding patient specific APM deficits is essential to realizing the full potential of restoration strategies in HNSCC.

## References

[1] Sung H Ferlay J Siegel RL Laversanne M Soerjomataram I Jemal A . Global cancer statistics 2020: GLOBOCAN estimates of incidence and mortality worldwide for 36 cancers in 185 countries. CA Cancer J Clin. (2021) 71:209–49. doi: 10.3322/caac.21660 33538338

[2] Chaturvedi AK Engels EA Pfeiffer RM Hernandez BY Xiao W Kim E . Human papillomavirus and rising oropharyngeal cancer incidence in the United States. J Clin Oncol. (2011) 29:4294–301. doi: 10.1200/JCO.2011.36.4596 21969503 PMC3221528

[3] Mehanna H Beech T Nicholson T El-Hariry I McConkey C Paleri V . Prevalence of human papillomavirus in oropharyngeal and nonoropharyngeal head and neck cancer—systematic review and meta-analysis of trends by time and region. Head Neck. (2013) 35:747–55. doi: 10.1002/hed.22015 22267298

[4] Burtness B Harrington KJ Greil R Soulières D Tahara M de Castro G . Pembrolizumab alone or with chemotherapy versus cetuximab with chemotherapy for recurrent or metastatic squamous cell carcinoma of the head and neck (KEYNOTE-048): a randomised, open-label, phase 3 study. Lancet Lond Engl. (2019) 394:1915–28. doi: 10.1016/S0140-6736(19)32591-7 31679945

[5] Bauml J Seiwert TY Pfister DG Worden F Liu SV Gilbert J . Pembrolizumab for platinum- and cetuximab-refractory head and neck cancer: results from a single-arm, phase II study. J Clin Oncol. (2017) 35:1542–9. doi: 10.1200/JCO.2016.70.1524 28328302 PMC5946724

[6] Chow LQM Haddad R Gupta S Mahipal A Mehra R Tahara M . Antitumor activity of pembrolizumab in biomarker-unselected patients with recurrent and/or metastatic head and neck squamous cell carcinoma: results from the phase Ib KEYNOTE-012 expansion cohort. J Clin Oncol. (2016) 34:3838–45. doi: 10.1200/JCO.2016.68.1478 27646946 PMC6804896

[7] Beatty GL Gladney WL . Immune escape mechanisms as a guide for cancer immunotherapy. Clin Cancer Res. (2015) 21:687–92. doi: 10.1158/1078-0432.CCR-14-1860 25501578 PMC4334715

[8] Kallingal A Olszewski M Maciejewska N Brankiewicz W Baginski M . Cancer immune escape: the role of antigen presentation machinery. J Cancer Res Clin Oncol. (2023) 149:8131–41. doi: 10.1007/s00432-023-04737-8 37031434 PMC10374767

[9] Ferris RL . Immunology and immunotherapy of head and neck cancer. J Clin Oncol. (2015) 33:3293–304. doi: 10.1200/JCO.2015.61.1509 26351330 PMC4586169

[10] Meissner M Reichert TE Kunkel M Gooding W Whiteside TL Ferrone S . Defects in the human leukocyte antigen class I antigen processing machinery in head and neck squamous cell carcinoma: association with clinical outcome. Clin Cancer Res. (2005) 11:2552–60. doi: 10.1158/1078-0432.CCR-04-2146 15814633

[11] Ogino T Shigyo H Ishii H Katayama A Miyokawa N Harabuchi Y . HLA class I antigen down-regulation in primary laryngeal squamous cell carcinoma lesions as a poor prognostic marker. Cancer Res. (2006) 66:9281–9. doi: 10.1158/0008-5472.CAN-06-0488 16982773

[12] Robbins Y Friedman J Redman J Sievers C Lassoued W Gulley JL . Tumor cell HLA class I expression and pathologic response following neoadjuvant immunotherapy for newly diagnosed head and neck cancer. Oral Oncol. (2023) 138:106309. doi: 10.1016/j.oraloncology.2023.106309 36682187 PMC9974754

[13] Little CC Tyzzer EE . Further experimental studies on the inheritance of susceptibility to a transplantable tumor, carcinoma (J. W. A.) of the Japanese waltzing mouse. J Med Res. (1916) 33:393–453 19972275 PMC2083849

[14] Snell GD . Methods for the study of histocompatibility genes. J Genet. (1948) 49:87–108. doi: 10.1007/BF02986826 18893744

[15] Zinkernagel RM Doherty PC . Restriction of *in vitro* T cell-mediated cytotoxicity in lymphocytic choriomeningitis within a syngeneic or semiallogeneic system. Nature. (1974) 248:701–2. doi: 10.1038/248701a0 4133807

[16] Townsend AR Rothbard J Gotch FM Bahadur G Wraith D McMichael AJ . The epitopes of influenza nucleoprotein recognized by cytotoxic T lymphocytes can be defined with short synthetic peptides. Cell. (1986) 44:959–68. doi: 10.1016/0092-8674(86)90019-x 2420472

[17] Neefjes JJ Momburg F Hämmerling GJ . Selective and ATP-dependent translocation of peptides by the MHC-encoded transporter. Science. (1993) 261:769–71. doi: 10.1126/science.8342042 8342042

[18] Rock KL Gramm C Rothstein L Clark K Stein R Dick L . Inhibitors of the proteasome block the degradation of most cell proteins and the generation of peptides presented on MHC class I molecules. Cell. (1994) 78:761–71. doi: 10.1016/S0092-8674(94)90462-6 8087844

[19] Ortmann B Copeman J Lehner PJ Sadasivan B Herberg JA Grandea AG . A critical role for tapasin in the assembly and function of multimeric MHC class I-TAP complexes. Science. (1997) 277:1306–9. doi: 10.1126/science.277.5330.1306 9271576

[20] Coux O Tanaka K Goldberg AL . Structure and functions of the 20S and 26S proteasomes. Annu Rev Biochem. (1996) 65:801–47. doi: 10.1146/annurev.bi.65.070196.004101 8811196

[21] Löwe J Stock D Jap B Zwickl P Baumeister W Huber R . Crystal structure of the 20 S proteasome from the archaeon T. acidophilum at 3.4 Å resolution. Science. (1995) 268:533–9. doi: 10.1126/science.7725097 7725097

[22] Rock KL Goldberg AL . Degradation of cell proteins and the generation of MHC class I-presented peptides. Annu Rev Immunol. (1999) 17:739–79. doi: 10.1146/annurev.immunol.17.1.739 10358773

[23] Ehring B Meyer TH Eckerskorn C Lottspeich F Tampé R . Effects of major‐histocompatibility‐complex‐encoded subunits on the peptidase and proteolytic activities of human 20S proteasomes: cleavage of proteins and antigenic peptides. Eur J Biochem. (1996) 235:404–15. doi: 10.1111/j.1432-1033.1996.00404.x 8631360

[24] Kisselev AF Akopian TN Woo KM Goldberg AL . The sizes of peptides generated from protein by mammalian 26 and 20 S proteasomes. J Biol Chem. (1999) 274:3363–71. doi: 10.1074/jbc.274.6.3363 9920878

[25] Garboczi DN Ghosh P Utz U Fan QR Biddison WE Wiley DC . Structure of the complex between human T-cell receptor, viral peptide and HLA-A2. Nature. (1996) 384:134–41. doi: 10.1038/384134a0 8906788

[26] Kelly A Powis SH Kerr LA Mockridge I Elliott T Bastin J . Assembly and function of the two ABC transporter proteins encoded in the human major histocompatibility complex. Nature. (1992) 355:641–6. doi: 10.1038/355641a0 1538751

[27] Nijenhuis M Hämmerling GJ . Multiple regions of the transporter associated with antigen processing (TAP) contribute to its peptide binding site. J Immunol. (1996) 157:5467–77. doi: 10.4049/jimmunol.157.12.5467 8955196

[28] Androlewicz MJ Anderson KS Cresswell P . Evidence that transporters associated with antigen processing translocate a major histocompatibility complex class I-binding peptide into the endoplasmic reticulum in an ATP-dependent manner. Proc Natl Acad Sci. (1993) 90:9130–4. doi: 10.1073/pnas.90.19.9130 8415666 PMC47515

[29] Alberts P Daumke O Deverson EV Howard JC Knittler MR . Distinct functional properties of the TAP subunits coordinate the nucleotide-dependent transport cycle. Curr Biol. (2001) 11:242–51. doi: 10.1016/S0960-9822(01)00073-2 11250152

[30] Kaklamanis L Leek R Koukourakis M Gatter KC Harris AL . Loss of transporter in antigen processing 1 transport protein and major histocompatibility complex class I molecules in metastatic versus primary breast cancer. Cancer Res. (1995) 55:5191–4 7585572

[31] Madden DR Gorga JC Strominger JL Wiley DC . The structure of HLA-B27 reveals nonamer self-peptides bound in an extended conformation. Nature. (1991) 353:321–5. doi: 10.1038/353321a0 1922337

[32] Vitiello A Potter TA Sherman LA . The role of β_2_ -microglobulin in peptide binding by class I molecules. Science. (1990) 250:1423–6. doi: 10.1126/science.2124002 2124002

[33] Sadasivan B Lehner PJ Ortmann B Spies T Cresswell P . Roles for calreticulin and a novel glycoprotein, tapasin, in the interaction of MHC class I molecules with TAP. Immunity. (1996) 5:103–14. doi: 10.1016/S1074-7613(00)80487-2 8769474

[34] Tector M Salter RD . Calnexin influences folding of human class I histocompatibility proteins but not their assembly with β2-microglobulin. J Biol Chem. (1995) 270:19638–42. doi: 10.1074/jbc.270.33.19638 7642652

[35] Hughes EA Cresswell P . The thiol oxidoreductase ERp57 is a component of the MHC class I peptide-loading complex. Curr Biol. (1998) 8:709–13. doi: 10.1016/S0960-9822(98)70278-7 9637923

[36] Bangia N Lehner PJ Hughes EA Surman M Cresswell P . The N-terminal region of tapasin is required to stabilize the MHC class I loading complex. Eur J Immunol. (1999) 29:1858–70. doi: 10.1002/(SICI)1521-4141(199906)29:06<1858::AID-IMMU1858>3.0.CO;2-C 10382748

[37] Dick TP Bangia N Peaper DR Cresswell P . Disulfide bond isomerization and the assembly of MHC class I-peptide complexes. Immunity. (2002) 16:87–98. doi: 10.1016/S1074-7613(02)00263-7 11825568

[38] Spiliotis ET Osorio M Zúñiga MC Edidin M . Selective export of MHC class I molecules from the ER after their dissociation from TAP. Immunity. (2000) 13:841–51. doi: 10.1016/S1074-7613(00)00081-9 11163199

[39] Wichmann G Vetter N Lehmann C Landgraf R Doxiadis I Großmann R . Outcome differences in HPV-driven head and neck squamous cell carcinoma attributable to altered human leukocyte antigen frequencies. Front Oncol. (2023) 13:1212454. doi: 10.3389/fonc.2023.1212454 38192630 PMC10772155

[40] Hopkins JR MacLachlan BJ Harper S Sewell AK Cole DK . Unconventional modes of peptide–HLA-I presentation change the rules of TCR engagement. Discov Immunol. (2022) 1:kyac001. doi: 10.1093/discim/kyac001 38566908 PMC10917088

[41] Rasmussen M Harndahl M Stryhn A Boucherma R Nielsen LL Lemonnier FA . Uncovering the peptide-binding specificities of HLA-C: a general strategy to determine the specificity of any MHC class I molecule. J Immunol. (2014) 193:4790–802. doi: 10.4049/jimmunol.1401689 25311805 PMC4226424

[42] Nagarsheth NB Norberg SM Sinkoe AL Adhikary S Meyer TJ Lack JB . TCR-engineered T cells targeting E7 for patients with metastatic HPV-associated epithelial cancers. Nat Med. (2021) 27:419–25. doi: 10.1038/s41591-020-01225-1 33558725 PMC9620481

[43] Maggs L Sadagopan A Moghaddam AS Ferrone S . HLA class I antigen processing machinery defects in antitumor immunity and immunotherapy. Trends Cancer. (2021) 7:1089–101. doi: 10.1016/j.trecan.2021.07.006 34489208 PMC8651070

[44] Mandal R Şenbabaoğlu Y Desrichard A Havel JJ Dalin MG Riaz N . The head and neck cancer immune landscape and its immunotherapeutic implications. JCI Insight. (2016) 1:e89829. doi: 10.1172/jci.insight.89829 27777979 PMC5070962

[45] Sun X Watanabe T Oda Y Shen W Ahmad A Ouda R . Targeted demethylation and activation of NLRC5 augment cancer immunogenicity through MHC class I. Proc Natl Acad Sci. (2024) 121:e2310821121. doi: 10.1073/pnas.2310821121 38300873 PMC10861931

[46] Yoshihama S Roszik J Downs I Meissner TB Vijayan S Chapuy B . NLRC5/MHC class I transactivator is a target for immune evasion in cancer. Proc Natl Acad Sci. (2016) 113:5999–6004. doi: 10.1073/pnas.1602069113 27162338 PMC4889388

[47] Zhou L Mudianto T Ma X Riley R Uppaluri R . Targeting EZH2 enhances antigen presentation, antitumor immunity, and circumvents anti–PD-1 resistance in head and neck cancer. Clin Cancer Res. (2020) 26:290–300. doi: 10.1158/1078-0432.CCR-19-1351 31562203 PMC6942613

[48] Ferris RL Whiteside TL Ferrone S . Immune escape associated with functional defects in antigen-processing machinery in head and neck cancer. Clin Cancer Res. (2006) 12:3890–5. doi: 10.1158/1078-0432.CCR-05-2750 16818683

[49] Leibowitz MS Andrade Filho PA Ferrone S Ferris RL . Deficiency of activated STAT1 in head and neck cancer cells mediates TAP1-dependent escape from cytotoxic T lymphocytes. Cancer Immunol Immunother. (2011) 60:525–35. doi: 10.1007/s00262-010-0961-7 21207025 PMC3426276

[50] Kinney BLC Gunti S Kansal V Parrish CJ Saba NF Teng Y . Rescue of NLRC5 expression restores antigen processing machinery in head and neck cancer cells lacking functional STAT1 and p53. Cancer Immunol Immunother. (2024) 73:10. doi: 10.1007/s00262-023-03589-y 38231444 PMC10794329

[51] Chandrasekaran S Sasaki M Scharer CD Kissick HT Patterson DG Magliocca KR . Phosphoinositide 3-kinase signaling can modulate MHC class I and II expression. Mol Cancer Res. (2019) 17:2395–409. doi: 10.1158/1541-7786.MCR-19-0545 31548239 PMC7339488

[52] Miyauchi S Kim SS Jones RN Zhang L Guram K Sharma S . Human papillomavirus E5 suppresses immunity via inhibition of the immunoproteasome and STING pathway. Cell Rep. (2023) 42:112508. doi: 10.1016/j.celrep.2023.112508 37171962 PMC10789500

[53] Heath BR Gong W Taner HF Broses L Okuyama K Cheng W . Saturated fatty acids dampen the immunogenicity of cancer by suppressing STING. Cell Rep. (2023) 42:112303. doi: 10.1016/j.celrep.2023.112303 36952341 PMC10514241

[54] Leibowitz MS Srivastava RM Andrade Filho PA Egloff AM Wang L Seethala RR . SHP2 is overexpressed and inhibits pSTAT1-mediated APM component expression, T-cell attracting chemokine secretion, and CTL recognition in head and neck cancer cells. Clin Cancer Res. (2013) 19:798–808. doi: 10.1158/1078-0432.CCR-12-1517 23363816 PMC3578140

[55] Ludigs K Seguín-Estévez Q Lemeille S Ferrero I Rota G Chelbi S . NLRC5 exclusively transactivates MHC class I and related genes through a distinctive SXY module. PloS Genet. (2015) 11:e1005088. doi: 10.1371/journal.pgen.1005088 25811463 PMC4374748

[56] Meissner TB Liu YJ Lee KH Li A Biswas A Van Eggermond MCJA . NLRC5 cooperates with the RFX transcription factor complex to induce MHC class I gene expression. J Immunol. (2012) 188:4951–8. doi: 10.4049/jimmunol.1103160 22490869 PMC3345046

[57] Wang B Niu D Lai L Ren EC . p53 increases MHC class I expression by upregulating the endoplasmic reticulum aminopeptidase ERAP1. Nat Commun. (2013) 4:2359. doi: 10.1038/ncomms3359 23965983 PMC3759077

[58] Zhu K Wang J Zhu J Jiang J Shou J Chen X . p53 induces TAP1 and enhances the transport of MHC class I peptides. Oncogene. (1999) 18:7740–7. doi: 10.1038/sj.onc.1203235 10618714

[59] The Cancer Genome Atlas Network . Comprehensive genomic characterization of head and neck squamous cell carcinomas. Nature. (2015) 517:576–82. doi: 10.1038/nature14129 25631445 PMC4311405

[60] Khalil MI Yang C Vu L Chadha S Nabors H James CD . The membrane-associated ubiquitin ligase MARCHF8 stabilizes the human papillomavirus oncoprotein E7 by degrading CUL1 and UBE2L3 in head and neck cancer. J Virol. (2024) 98:e01726-23. doi: 10.1128/jvi.01726-23 38226814 PMC10878100

[61] Liao TT Chen YH Li ZY Hsiao AC Huang YL Hao RX . Hypoxia-induced long noncoding RNA HIF1A- AS2 regulates stability of MHC class I protein in head and neck cancer. Cancer Immunol Res. (2024) 12:1468–84. doi: 10.1158/2326-6066.CIR-23-0622 38920249 PMC11443317

[62] Morris LGT . Loss of human leukocyte antigen and immune escape in head and neck cancer. Laryngoscope. (2024) 134:160–5. doi: 10.1002/lary.30761 37249223 PMC10687312

[63] Cornel AM Mimpen IL Nierkens S . MHC class I downregulation in cancer: underlying mechanisms and potential targets for cancer immunotherapy. Cancers. (2020) 12:1760. doi: 10.3390/cancers12071760 32630675 PMC7409324

[64] Ayers M Lunceford J Nebozhyn M Murphy E Loboda A Kaufman DR . IFN-γ–related mRNA profile predicts clinical response to PD-1 blockade. J Clin Invest. (2017) 127:2930–40. doi: 10.1172/JCI91190 28650338 PMC5531419

[65] Hélène C Conrad O Pflumio C Borel C Voegelin M Bernard A . Dynamic profiling of immune microenvironment during anti-PD-1 immunotherapy for head and neck squamous cell carcinoma: the IPRICE study. BMC Cancer. (2023) 23:1209. doi: 10.1186/s12885-023-11672-x 38066522 PMC10704641

[66] Pestka S Kotenko SV Muthukumaran G Izotova LS Cook JR Garotta G . The interferon gamma (IFN-γ) receptor: a paradigm for the multichain cytokine receptor. Cytokine Growth Factor Rev. (1997) 8:189–206. doi: 10.1016/S1359-6101(97)00009-9 9462485

[67] Srivastava RM Trivedi S Concha-Benavente F Hyun-Bae J Wang L Seethala RR . STAT1-induced HLA class I upregulation enhances immunogenicity and clinical response to anti-EGFR mAb cetuximab therapy in HNC patients. Cancer Immunol Res. (2015) 3:936–45. doi: 10.1158/2326-6066.CIR-15-0053 25972070 PMC4526378

[68] Concha-Benavente F Ferris RL . IFNγ-induced PD-L1 expression is JAK2 but not JAK1 dependent and its inhibition enhances NK-cetuximab mediated ADCC of HNSCC cells. J Immunother Cancer. (2014) 2:2051-1426-2-S3-P199. doi: 10.1186/2051-1426-2-S3-P199 38164791

[69] Zhang S Zheng M Nie D Xu L Tian H Wang M . Efficacy of cetuximab plus PD-1 inhibitor differs by HPV status in head and neck squamous cell carcinoma: a systematic review and meta-analysis. J Immunother Cancer. (2022) 10:e005158. doi: 10.1136/jitc-2022-005158 36253001 PMC9577924

[70] Harrington KJ Cohen EEW Soulières D Dinis J Licitra L Ahn MJ . Pembrolizumab versus methotrexate, docetaxel, or cetuximab in recurrent or metastatic head and neck squamous cell carcinoma (KEYNOTE-040): subgroup analysis by pattern of disease recurrence. Oral Oncol. (2023) 147:106587. doi: 10.1016/j.oraloncology.2023.106587 37925894

[71] Verma AK Messerli SM Miskimins WK . Lactate induces PD-L1 in HRASG12V-positive oropharyngeal squamous cell carcinoma. Oncotarget. (2020) 11:1493–504. doi: 10.18632/oncotarget.27348 32391119 PMC7197448

[72] Wang S He Z Wang X Li H Liu XS . Antigen presentation and tumor immunogenicity in cancer immunotherapy response prediction. eLife. (2019) 8:e49020. doi: 10.7554/eLife.49020 31767055 PMC6879305

[73] Takeuchi M Doi T Obayashi K Hirai A Yoneda K Tanaka F . Soluble PD-L1 with PD-1-binding capacity exists in the plasma of patients with non-small cell lung cancer. Immunol Lett. (2018) 196:155–60. doi: 10.1016/j.imlet.2018.01.007 29366663

[74] Ando F Kashiwada T Kuroda S Fujii T Takano R Miyabe Y . Combination of plasma MMPs and PD-1-binding soluble PD-L1 predicts recurrence in gastric cancer and the efficacy of immune checkpoint inhibitors in non-small cell lung cancer. Front Pharmacol. (2024) 15:1384731. doi: 10.3389/fphar.2024.1384731 38774209 PMC11106465

[75] Schmitt NC Trivedi S Ferris RL . STAT1 activation is enhanced by cisplatin and variably affected by EGFR inhibition in HNSCC cells. Mol Cancer Ther. (2015) 14:2103–11. doi: 10.1158/1535-7163.MCT-15-0305 26141950 PMC4561003

[76] Reits EA Hodge JW Herberts CA Groothuis TA Chakraborty M K.Wansley E . Radiation modulates the peptide repertoire, enhances MHC class I expression, and induces successful antitumor immunotherapy. J Exp Med. (2006) 203:1259–71. doi: 10.1084/jem.20052494 16636135 PMC3212727

[77] Villa A Sonis S . Toxicities associated with head and neck cancer treatment and oncology-related clinical trials. Curr Probl Cancer. (2016) 40:244–57. doi: 10.1016/j.currproblcancer.2016.06.001 27520848

[78] McMasters KM Egger ME Edwards MJ Ross MI Reintgen DS Noyes RD . Final results of the Sunbelt Melanoma Trial: a multi-institutional prospective randomized phase III study evaluating the role of adjuvant high-dose interferon alfa-2b and completion lymph node dissection for patients staged by sentinel lymph node biopsy. J Clin Oncol. (2016) 34:1079–86. doi: 10.1200/JCO.2015.63.3776 26858331 PMC5321066

[79] Kalbasi A Tariveranmoshabad M Hakimi K Kremer S Campbell KM Funes JM . Uncoupling interferon signaling and antigen presentation to overcome immunotherapy resistance due to JAK1 loss in melanoma. Sci Transl Med. (2020) 12:eabb0152. doi: 10.1126/scitranslmed.abb0152 33055240 PMC8053376

[80] Ye W Gunti S Allen CT Hong Y Clavijo PE Van Waes C . ASTX660, an antagonist of cIAP1/2 and XIAP, increases antigen processing machinery and can enhance radiation-induced immunogenic cell death in preclinical models of head and neck cancer. OncoImmunology. (2020) 9:1710398. doi: 10.1080/2162402X.2019.1710398 32002309 PMC6959437

[81] Bourhis J Licitra LF Burtness B Psyrri A Haddad R Harrington K . Xevinapant or placebo plus platinum-based chemoradiotherapy in unresected locally advanced squamous cell carcinoma of the head and neck (TrilynX): a randomized, phase III study. J Clin Oncol. (2025) 43:3209–20. doi: 10.1200/JCO-25-00272 40902136 PMC12509437

[82] Chen NX Liu K Liu X Zhang XX Han D . Induction and regulation of the immunoproteasome subunit β5i (PSMB8) in laryngeal and hypopharyngeal carcinoma cells. Med Sci Monit. (2020) 26:e923621. doi: 10.12659/MSM.923621 32680979 PMC7366787

[83] Chakraborty AK Kroehling L Raut RD Choudhury C Kukuruzinska MA Gutkind JS . LSD1 inhibition induces MHC-I and dendritic cell activation to promote antitumor immunity in head and neck squamous cell carcinoma. Cancer Res. (2026) 86:503–18. doi: 10.1158/0008-5472.CAN-24-3239 41066547 PMC12599148

[84] Song J Yang P Chen C Ding W Tillement O Bai H . Targeting epigenetic regulators as a promising avenue to overcome cancer therapy resistance. Signal Transduct Target Ther. (2025) 10:219. doi: 10.1038/s41392-025-02266-z 40675967 PMC12271501

[85] Maddineni S Silberstein JL Sunwoo JB . Emerging NK cell therapies for cancer and the promise of next generation engineering of iPSC-derived NK cells. J Immunother Cancer. (2022) 10:e004693. doi: 10.1136/jitc-2022-004693 35580928 PMC9115029

[86] Lee MY Robbins Y Sievers C Friedman J Abdul Sater H Clavijo PE . Chimeric antigen receptor engineered NK cellular immunotherapy overcomes the selection of T-cell escape variant cancer cells. J Immunother Cancer. (2021) 9:e002128. doi: 10.1136/jitc-2020-002128 33741731 PMC7986659

[87] Moreno-Nieves UY Tay JK Saumyaa S Horowitz NB Shin JH Mohammad IA . Landscape of innate lymphoid cells in human head and neck cancer reveals divergent NK cell states in the tumor microenvironment. Proc Natl Acad Sci. (2021) 118:e2101169118. doi: 10.1073/pnas.2101169118 34244432 PMC8285950

[88] Puram SV Tirosh I Parikh AS Patel AP Yizhak K Gillespie S . Single-cell transcriptomic analysis of primary and metastatic tumor ecosystems in head and neck cancer. Cell. (2017) 171:1611–1624.e24. doi: 10.1016/j.cell.2017.10.044 29198524 PMC5878932

[89] Ruffin AT Li H Vujanovic L Zandberg DP Ferris RL Bruno TC . Improving head and neck cancer therapies by immunomodulation of the tumour microenvironment. Nat Rev Cancer. (2023) 23:173–88. doi: 10.1038/s41568-022-00531-9 36456755 PMC9992112

[90] Zwing N Voith Von Voithenberg L Alberti L Gabriel SM Monné Rodriguez JM Feddersen R . Mapping immune activity in HPV-negative head and neck squamous cell carcinoma: a spatial multiomics analysis. J Immunother Cancer. (2025) 13:e011851. doi: 10.1136/jitc-2025-011851 40562703 PMC12198833

[91] Santharam MA Shukla A Levesque D Kufer TA Boisvert FM Ramanathan S . NLRC5-CIITA fusion protein as an effective inducer of MHC-I expression and antitumor immunity. Int J Mol Sci. (2023) 24:7206. doi: 10.3390/ijms24087206 37108368 PMC10138588

[92] Sun X Zhang Y Li J Park KS Han K Zhou X . Amplifying STING activation by cyclic dinucleotide–manganese particles for local and systemic cancer metalloimmunotherapy. Nat Nanotechnol. (2021) 16:1260–70. doi: 10.1038/s41565-021-00962-9 34594005 PMC8595610

[93] Chaudhary S Dam V Ganguly K Sharma S Atri P Chirravuri-Venkata R . Differential mutation spectrum and immune landscape in African Americans versus Whites: a possible determinant to health disparity in head and neck cancer. Cancer Lett. (2020) 492:44–53. doi: 10.1016/j.canlet.2020.07.029 32738272 PMC8432304

[94] Yang Y Chen L Shang M Cao Q Zhang M Zhang L . Integrated analysis of sex differences in human dendritic cell, monocyte, and natural killer cell subsets. Front Immunol. (2026) 17:1750775. doi: 10.3389/fimmu.2026.1750775 41983128 PMC13070752

[95] Jagadeesha S Lamenza F Upadhaya P Chung C Webb A Nyati K . Sex-based immune differences influence tumor progression in head and neck squamous cell carcinoma. Oral Oncol. (2026) 176:107929. doi: 10.1016/j.oraloncology.2026.107929 41818928 PMC13075524

[96] Thasma Loganathan VK Satishkartik S Pattabiram S Suresh Kumar A Chattopadhyay S Karthikeyan V . RNA cargo in motion: the exosomal connection to head and neck cancers. ExRNA. (2025) 7 (1):0003. doi: 10.55092/exrna20250003

